# Healthy Even through Cancer: What Are the Assumptions and Outcomes for Psychological Intervention?

**DOI:** 10.3390/bs13060506

**Published:** 2023-06-16

**Authors:** Eleonora Pinto

**Affiliations:** Unit of Surgical Oncology of the Esophagus and Digestive Tract, Veneto Institute of Oncology IOV—IRCCS, 35128 Padua, Italy; eleonora.pinto@iov.veneto.it

## 1. Introduction

Cancer remains a highly fatal disease, a major cause of mortality and a huge health burden around the world, requiring increased primary prevention efforts, screenings and treatments [1]. When considering neoplasm disease, the side effects of treatments and iatrogenesis should also be taken into account in order to estimate patients’ health on a global scale.

Cancer is globally considered a threat that needs to be controlled [1]: the global burden of disease, accounting for cancer incidence, mortality, years lived with disability, years of life lost, and disability-adjusted life-years, demonstrates a broad and heterogeneous cancer spread worldwide, highlighting 24.5 million cancer cases in 2017. Moreover, in 2017, there were 9.6 million cancer deaths. Even though the current oncological studies can differentiate between overall survival, disease-specific survival and disease-free survival, providing indicators of the improvement in treatment outcomes, of the whole amount of disability-adjusted life-years caused by cancer, it was estimated that the 97% came from years of life lost, while only the 3% came from years lived with disability [1].

Therefore, it is globally recognized that health cannot be recognized as a disease-free condition, nor have adaptative attempts been made, since health does not match a sole body condition and due to related variability of physiological adaptation; health can instead be recognized as the modality used by individuals in managing their disease [2]. Specifically, health can be described as a process entailing the possibility of narrating and encompassing the interaction between the patient, the neoplasm and the health professional aimed at treating it [2].

In this sense, in current oncological settings and in the related literature, not only biological parameters but also quality of life have become the main outcomes evaluated in order to assess treatment, to screen patients, to detect morbidity and to predict survival [3]. This self-reported outcome is a benchmark of the increasing need to “be healthy”, through experiencing the disease even before the achievement of oncological treatment goals or despite their failure. Therefore, health professionals should construct a treatment pathway for patients’ health through the neoplasm.

In this context, psychological interventions have become part of the multidisciplinary, integrated care pathway. Thus, some questions are crucial for providing an appropriate psychological contribution: (1) what are the objectives of psychological support for oncological patients? (2) What are psychological interventions specific for cancer patients based on? (3) What are the outcomes of psychological interventions?

The following contributions presented in the Special Issue entitled “Healthy Even through Cancer: What Are the Assumptions and Outcomes for Psychological Intervention?” aim to provide a picture of the state-of-the-art of psychological support in oncology, answering the above-mentioned key questions for clinicians.

## 2. What Are the Objectives of Psychological Support for Oncological Patients?

The research conducted points out needs identification as the mandatory step in order to detect specific aims. Broadly, subsuming the bodily dimension of health [4,5,6,7,8], the need to deepen observations regarding how physical impairments and different scenarios are outlined can be identified. So, generally in the presented manuscripts, the global dimension of health in individuals suffering from cancer is stated as being subject to overwhelming contradictions of events and reflections, of facts and reports on such facts. Additionally, the need to synthesize new meanings and promote the organization and connection of different elements of experience is stressed [9,10,11,12]. Throughout these needs, involving all of the actors with specific aims, roles and contributions to the treatment of cancer in different ways is considered relevant overall [4,5,6,13].

In particular, in “The Health of Healthcare Professionals in Italian Oncology: An Analysis of Narrations through the M.A.D.I.T. Methodology” [4], the research aim is describing the discursive modalities used by the roles that contribute to generating the configuration of the reality of the “health of the health worker who works with cancer patients” (operators, patients and the affective nucleus, citizens).

In “Managing the Consequences of Oncological Major Surgery: A Short- and Medium-Term Skills Assessment Proposal for Patient and Caregiver through M.A.D.I.T. Methodology”, the aim is to assess patients’ competences in postoperative management [5].

In “Critical Competences for the Management of Post-Operative Course in Patients with Digestive Tract Cancer: The Contribution of MADIT Methodology for a Nine-Month Longitudinal Study” [6], the aim is implementing competencies in GI and upper GI patients having undergone surgery.

In “A Textual Analysis for Understanding the Relations and the Identity Construction in Adolescent Oncology Patients: Retrospective Personal Views in Order to Educate Health Professionals” [8], the researchers describe the following sub-objectives: establishing how adolescent cancer patients (at the end of treatment or in the period of follow-up) identify the specifics of doctor–patient communication during the treatment of adolescent patients in follow up; identifying fears, thoughts, and changes in self-perception; identifying the role of loved ones in relation to treatment; and identifying whether respondents had an active role in managing their treatment path.

In “Experiences of Female Breast Cancer Survivors Concerning Their Return to Work in Spain” [9], the researchers aimed to analyze the experience of returning to work of women who had overcome breast cancer, identifying the sequent sub-objectives: outlining the degree of physical and psychological consequences from cancer; identifying the personal motivation to return to work; describing the options and possible welfare and working facilities available; and establishing the difficulties encountered by patients.

In “Anticipatory Mourning and Narrative Meaning-Making in the Younger Breast Cancer Experience: An Application of the Meaning of Loss Codebook” [10], the authors explore coping strategies, perceived resources, meanings attributed to illness and loss and possible perceived growth.

In “How to Intervene in the Health Management of the Oncological Patient and of Their Caregiver? A Narrative Review in the Psycho-Oncology Field” [7], the objective is to describe the state of the art of the psychological treatment for patients and their caregivers, from diagnosis to follow-up.

The case report “Mindfulness Meditation as Psychosocial Support in the Breast Cancer Experience: A Case Report” [11] describes the clinical path of a patient with breast cancer. It specifically aims to encourage the resumption of social and working life roles and to rebuild a new identity, characterized by awareness, acceptance and personal growth.

Another case report, “The Role of the Psycho-Oncologist during the COVID-19 Pandemic: A Clinical Breast Cancer Case Report” [12], focuses on the critical role of intervention and psychological support during the COVID-19 pandemic in the cancer context. It aims to describe coping skills facing the neoplasm during an emergency and its consequences (for example, a sense of vulnerability, loneliness, and helplessness, fear, anxiety, distress, perception of responsibility).

Finally, “The Co-Construction of an Elegant Ending—Polyphonic Musical Intervention in Palliative Care: A Case Study” [13] is based on the need to trigger “interpersonal awareness”, and it aims to deepen the redesigning palliative care method rooted in polyphonic exercises.

In general, all of the needs presented in these authors’ works lie in promoting a shift from the use of deterministic-causal criteria to the management of the interactions in oncological settings and the interaction with uncertainty caused by the neoplasm. From these needs, the objective can be globally set as improving the interactive modalities and strategies of constructing a healthcare path when a neoplasm is diagnosed.

## 3. What Are Psychological Interventions Specific for Cancer Patients Based on?

The interventions carried out in the papers presented here show different theoretical assumptions for psychological interventions in oncology. Across the contributions presented, with different levels of details, the interventions are based on language: language analysis can provide indications for psychological strategies and intervention.

For example, process and content analysis is needed to outline the use-value of patient’s language [4,5,6,8]. Both themes and contents, methodologically defined as archipelagos of meaning emerging from texts and the discursive modalities through which the content is offered in the text, are considered. These contributions are grounded in dialogical science which studies interactive dialogical processes in natural language generating configurations of realities [2] and the rules of use of natural language. Natural language-ordinary language analysis allows for the description of processes on the basis of the rhetorical–argumentative links. Dialogical science identifies the M.A.D.I.T. (Methodology for the Analysis of Computerized Textual Data) methodology [14,15] as coherent and well founded, since it belongs to the same epistemological basis as dialogical science. In M.A.D.I.T., the discursive modalities are referred to as what dialogic science calls discursive repertories, whose definition and measurement authors present [4], composing the reality under observation. Three types of discursive repertoires and related rules of the natural language are detected: generative, stabilization and hybrid repertories. So, through M.A.D.I.T., the variability of dialogical processes and different senses of reality are described. Moreover, authors point out that the modalities by which patients report any condition, the diagnosis, the treatment, the symptoms, how patients give their self-description or describe other roles can lead to understanding patients’ representations. suggesting that this methodology makes a personalized approach possible [5], based on data derived from the particular discourses used by patients.

Other contributions [9,10] stress the primary role of language in conveying meaning. They assume that meanings are embodied in words and so engage in a consistent interlocution with theory (for example, by means of a bottom-up approach). In “Anticipatory Mourning and Narrative Meaning-Making in the Younger Breast Cancer Experience: An Application of the Meaning of Loss Codebook” [10], the authors apply the categories related to the meaning of loss codebook to the narratives of women affected by breast cancer in a top-down and bottom-up study. In particular, in the authors’ proposal, the meaning of loss codebook can be considered a narrative exploratory tool for observing meanings in the course of treatment and how their construction changes. The method used is the narrative interview, with open questions collecting episodic and semantic narratives, with each question intended as a narrative prompt designed to open up a construction of sense at each stage of the ongoing experience and designed to activate different ways of describing the narrative discourse [10].

Similarly, in “Experiences of Female Breast Cancer Survivors Concerning Their Return to Work in Spain” [9], the authors choose the focus group as a strategy to collect narrations and select an appropriate qualitative analysis tool (Atlas.ti) to organize, store, compare, and analyze the data obtained in the interviews, through the consensus of researchers. In this research, the method encompasses content analysis, based on patients’ words and expressions following five explicated criteria (completeness, exclusivity, semi-induction, belonging to the category, objectivity).

The three case reports presented [11,12,13] extract pieces of text directly referring to words (verbatim) used by patients. Indeed, in these studies, the researchers explore the psychological and existential meanings, analyzing and interpreting narratives observed in order to achieve meanings based on patients’ experiences and values. Starting from this assumption, the researchers state that different techniques can be used to change experiences, for example mindfulness or polyphonic musical intervention.

Lastly, the examination of narrations presented in the current literature permits the focus on psychological intervention: inquiry to the literature is a necessary, preordered step. This aspect is reflected in other contributions in the background, but it is carried out in particular by “How to Intervene in the Health Management of the Oncological Patient and of Their Caregiver?” [7], which is a narrative review based on 68 articles, collecting the most relevant discourses and arguments regarding interventions.

## 4. What Are the Outcomes of Psychological Intervention?

In view of the needs and global aim observed in the manuscripts presented here and outlined above, the outcomes of interventions can be recognized not only amongst patients, but also among all of the roles involved in the care pathway: patients, healthcare professionals, and caregivers. Moreover, outcomes are related to representations given by these “relevant voices” through language.

These aspects are broadly shared in contributions involving family members and doctors as roles interacting with patients and so contributing to the generation of skills in disease coping. In particular, Turchi, Salvalaggio et al. [4] focus on the narrated reality as the outcome: narration is not merely “what has been said”, it is the sense of reality held in both contents and processes obtained through the textual analysis. In this study concerning health workers, three areas are specified: the first concerns the narrative reality of the healthcare professionals serving users with cancer, the second regards their health promotion and the third concern cancer patients’ health promotion. This study shows that the level of health in health professionals is low, and therefore intervening to broaden the possibility of these professionals narrating their professional role differently appears mandatory.

Additionally, Bomben et al. [8] highlight narratives as outcomes. Specifically, outcomes are distinguished in four macro areas: (1) the particulars of patient–physician communications regarding the disease; (2) patients’ thoughts, fears and changes in their self-perception; (3) “other meanings” shaped during the treatment pathway; and (4) modalities for reacting to the disease and its treatment. Bomben and collegues indicate the assumptions of post-modern psychology and the framework of the constructionist paradigm, according to which personal identity is constructed in a dialogical sense, within relational and hermeneutic contexts. This study stresses that language using expressions leading to an immutable framework causes patients to identity based only on the experience of illness, diverting resources away from their own health promotion [8].

Furthermore, this study shows that cancer in young adults and adolescents affects all domains of life: physical, psycho-emotional, and relational. Therefore, a complementary treatment to medical care, in a multidisciplinary approach focused on health and not only on the disease of the body, is suggested. Patient–physician communication, consideration of the whole network of relationships, and attention to patients’ reactions are also recommended by authors for compliance and adherence to treatment.

In Turchi, Fabbian et al. [5] study, the outcome is the use of particular competences, namely technical–operational modalities and the relationships acquired and developed in specific roles, namely the patient and caregiver. The same outcome is outlined in Pinto, Fabbian et al. [6]. These works point out that patients’ competencies are not determined by diagnosis, therapy or patients’ comorbidities, whilst they rely on particular language modalities prioritizing the evaluation of choices made in daily life during and after treatment, rather than the representation of future scenarios linked to postoperative conditions.

Achievements in this research point out low competency levels for both patients and caregivers. These low levels may be related to the representation of oncological surgery treatment as something that will change the disease, and the resulting complete delegation of health promotion to health professionals.

Moreover, the interventional study [6] explores how narratives can vary in neoplasm treatment management and, through data on the interventions carried out, suggests how these competences can be improved.

In the study conducted by Turchi, Dalla Riva et al. [7], outcomes are organized on the basis of the information that emerged about the patient, caregiver, patient–caregiver dyad and health-professionals. The description of the review’s results leads the authors to identify the need for assessing a common health definition and providing related interventions to support the roles of the community involved in oncological diagnosis. In particular, this description implies that establishing the most suitable and pertinent language is mandatory in research, especially in evaluation studies concerning the effectiveness of psychological interventions.

Dealing with job loss and the return to the workplace, Aguiar-Fernández and colleagues [9] explore patients’ interpretations of their experiences through qualitative analysis. Using three themes (self-perceptions of cancer survivors; motivation to go back to work; and going back to work: the pathways and difficulties) further specified “into secondary categories according to their level of depth” [6], in this study, rhetoricals, patients’ observational categories, can be identified. Indeed, the qualitative results allow for obtaining the participants’ perspectives and points of view (their emotions, priorities, meanings, etc.).

For these authors, intervening in treatment consequences is strategic in order to promote patients’ job resumption, since based on this study’s exploration, there is a lack of legal and administrative support, in coordination between health and social services.

For Martino and colleagues [10], the study’s outcome is psychological adaptation to the specific characteristics of cancer as a stressor. This outcome needs to consider the uncertainty of a neoplasm characterized by unpredictability. As observed in other studies, in this research, the outcome is composed of some thematic areas described by the authors as meaning transformations during the different phases of treatment: coping, moving on (constructive response), acceptance, emotionality, negative affect and regret. The tool proposed in this study is also considered as a preventive tool, in order to observe the meanings’ inflexibility, allowing the recommendation of a psychological intervention aimed at supporting the flexibility of meaning in relation to a particular experience. Indeed, the flexibility of meaning in relation to cancer experience is supported by the authors’ observations.

The outcomes described by Iannopollo and colleagues in the case report regarding a mindfulness-based intervention [11] are the improvement of patients’ psycho-emotional state, self-criticism, isolation, insomnia and skills of mentalization, self-acceptance and evaluation of choices.

This study’s output is an improvement in patients’ quality of life (the patient resumes planning, sleep–wake rhythms are more regular, better management of symptoms related to the trauma of the disease) after group intervention combined with individual psychological interventions.

The case report from during the COVID-19 pandemic [12] achieves the aim of treating cancer with a multidisciplinary approach paying attention to physical, psychological and social aspects in each treatment step. Improving clinical outcomes is one of the objectives stated in a breast patient case study presented by Silvestri and colleagues [12] by means of analysis and intervention on modalities used for facing criticalities and difficulties in oncological setting. Therefore, in this case, the outcome in the psychotherapist’s plan concerns the improvement of psycho-emotional relaxation (adaptation and maintenance), an adequate internal locus of control in the psychological adaptation to the oncological disease, and the promotion of a proactive experience towards the disease [12]. The authors state that the patient developed the skill of managing anxiety about the future and fear of recurrence in an adaptable and functional way in the pandemic context.

In the end-of-life experience contribution given by Schiavo [13], the outcome is seen as successful performance based on musical exercises and games, with the regulation of emotions, and therefore the language chosen by patients and caregivers can be verbal, prosodic, or based facial expressions, thus constructing the time close to death between the patient and caregiver together, sharing the same symbolic universe, its rules and its perlocutory implications [16].

Hence, all of the outcomes presented in the contributions are constructs, or rather symbolic units with particular values within particular theoretical fields, expressing a sense of reality not overlapping with clinical parameters or overwhelming them.

## 5. Conclusions

Some of the features of these contributions demonstrate them to be on a common line.

This line begins when discourses, narrations, competencies are described and explored and the methods and criteria taken into account are explained. All researchers are invited to define the specific object of study, explain the methodology, and analyze and present the indicators of psychological support. The interaction between patients and other roles involved in neoplasm management is considered across the contributions and, highlighting the improving competencies in each role, improves overall health.

Additionally, many studies presented here show coherence in terms of the referred constructs, even if not all of contributions present operational tools to detect criticalities. In this respect, when presented in the studies, the use of ad hoc tools promotes and facilitates communication between roles interacting with patients. In fact, both in clinical intervention and in research outcomes, the attention paid to the caregiver role and the family unit permits alignment between goals and the impact on the management of critical situations, thus corroborating the knowledge on cancer patient health. In detail, the outcomes presented in this Special Issue are qualitative as well as interactive, since the outcomes in oncological settings have to consider the neoplasms, treatments and treatment effects with which different roles interact.

Hence, these contributions demonstrate the need for change in the knowledge paradigm from a causalist one to a narrativistic one, where methods should be different. There is a need to go beyond the body level, considering the medical level as an instrument that should be used by patients to confirm the possibility of pursuing health, acting in terms of health, describing all of the healthy options even after a tumor diagnosis or during treatment. Furthermore, the configuration of constructs used by researchers can shared a common definition. Since all studies rely on narratives of relevant voices considered in the research focus, a health definition grounded in an adequate epistemological level, referred to as language, is required. Thus, the assumption is that health is “a configuration generated in the context of a dialogic process which foresees pathologies and/or implications of actions on the organic level as well as on the level of interaction in the community. There is not a normative ‘limit’ of a maximum attainable level of self-perceived health or a level completely divorced from health in which we cannot speak of health, indeed self-perceived health still continues to exist as a process” [2]. This assumption can help to define strategies in research and in clinical approaches.

Finally, the manuscripts in this Special Issue contribute to supporting qualitative methodologies and the related instruments used by researchers and clinicians, deepening the theoretical basis of language study in the psychoncological research field.

Consequently, all of these aspects suggest a direction for the effective integration of psychological interventions in cancer patient care: in this way, health promotion can be always a possible goal set and reached for oncological patients.
